# Global availability of guidelines related to assistive technology: a scoping review

**DOI:** 10.3389/fresc.2025.1581104

**Published:** 2025-04-24

**Authors:** Wei Zhang, Johan Borg

**Affiliations:** ^1^ Master Program of Global Health Leadership, Nuffield Department of Primary Care, Health Sciences, and Said Business School, University of Oxford, Oxfordshire, United Kingdom; ^2^ Department of Medical Sciences, School of Health and Welfare, Dalarna University, Falun, Sweden

**Keywords:** assistive technology, guidelines, health products, health system, health workforce, universal health coverage

## Abstract

**Background:**

Given the rising global demand for assistive technology, predicted to encompass 3.5 billion people by 2050, understanding the availability of guidelines governing its use and identifying potential gaps is paramount.

**Objective:**

This scoping review mapped existing guidelines related to assistive technology. The review aimed to inform future research and guideline development to accelerate access to assistive technology within universal health coverage.

**Methods:**

Following the JBI methodology, a systematic search of guidelines published between January 2008 and March 2024 was conducted across CINAHL, Google Scholar, PubMed, TRIP and WHO IRIS. Included guidelines related to specific assistive technology, including product types and services for users and their caregivers. Guidelines targeting system-level interventions were excluded.

**Results:**

The search identified 291 records, of which 24 guidelines were included. They focus on improving health outcomes for diverse populations across different healthcare settings. Most guidelines originated from high-income countries and predominantly addressed commonly known assistive products for mobility, hearing, vision, and self-care. There is a gap in guidelines for assistive products for cognition and communication. The identified guidelines primarily followed evidence-based methodologies and involved assistive technology users in their development.

**Conclusions:**

This review provides a crucial overview of the existing landscape of assistive technology guidelines. It calls for further action to harmonize standards, leverage innovation in evidence generation, and enhance guideline development to better serve the global population in need of assistive technology.

## 1 Introduction

Assistive technology refers to products and their related systems and services designed to enhance health and well-being, especially for individuals with disabilities, older adults, and those living with chronic health conditions (1). Assistive products include physical devices such as wheelchairs, spectacles, hearing aids, prostheses, and digital applications that support interpersonal communication and daily activities as well as environmental adaptations such as ramps and grab-rails (1, 2). According to the 2022 Global Report on Assistive Technology (3), around 2.5 billion people worldwide need at least one assistive product, a figure projected to rise to over 3.5 billion by 2050. However, access to assistive technology (i.e., people having the needed assistive products as a proportion of the population in need) varies significantly and is below 5% in some countries (3).

The 71st World Health Assembly resolution (WHA 71.8) called for Member States to develop, implement and strengthen policies and programs, to improve access to assistive technology within universal health and social services coverage (4). The resolution underscores the need for establishing national lists of priority assistive products that are affordable and meet minimum quality and safety standards.

While the International Organization for Standardization (ISO) has classified over 900 types of assistive products, providing an extensive reference for different health conditions (2), WHO introduced the Priority Assistive Products List (APL) in 2016 (1). Aiming to improve access to assistive technology globally, the APL prioritizes 50 products with widespread need and the greatest impact on people's lives. The ISO and WHO lists serve as resources, but the wide array of products and lack of guidance on implementing and monitoring their provision can challenge national adaptation and policymaking. Despite progress in country initiatives (5), many countries struggle to fully implement policies or monitor their impact due to noticeable workforce and service delivery gaps (3, 6).

Guidelines, according to World Health Organization, are any document containing recommendations for clinical practice or public health policy (7), are crucial for translating evidence into policies and practices for assistive technology. The global emphasis on improving access to assistive technology highlights the need for a clearer understanding of existing guidelines and areas for improvement. Despite numerous assistive products and a growing global demand, a gap remains in comprehensive, accessible guidance for policymakers and practitioners. A preliminary literature search conducted in July 2023, using terms related to “assistive products” and “guidelines” in databases such as PubMed and the Cochrane Database, revealed no comprehensive reviews on this topic. Thus, there is a need for a scoping review aiming to map existing guidelines related to assistive technology and to identify resources and gaps in available guidelines. It would support policymakers, practitioners, and researchers by offering an overview of existing guidelines and highlighting the gaps that require further exploration. By mapping the existing resources, a review would inform guideline developers, including the WHO and other stakeholders, on priority areas for future research and normative work in assistive technology. Addressing the existing gaps and understanding national and international best practices are essential for shaping effective, equitable policies that enhance accessibility and meet the needs of an aging, increasingly diverse population.

Consequently, the objective of this scoping review was to map existing guidelines related to assistive technology. The primary review question was: What guidelines related to assistive technology have been published globally? To provide a comprehensive understanding of the scope and characteristics of the identified guidelines, the following secondary review questions were addressed:

1. Which assistive products and related services are covered in the identified guidelines?
2. For which population groups (considering age, gender, geographic location, and health conditions or functional difficulties) are the identified guidelines intended?
3. In which contexts (such as home, community, primary care, or specialty care) are the identified guidelines applicable?
4. Were the identified guidelines developed following an established methodology?

## 2 Methods

### 2.1 Protocol and registration

This review followed the JBI methodology for scoping reviews (8). The review protocol was registered on 23 May 2024 in the Open Science Framework (Registration DOI: https://doi.org/10.17605/OSF.IO/FV7J8). The protocol was updated on 3 June 2024,^1^ revising the title of the review to focus on the global availability of the guidelines. This update was justified to better reflect the scope of the review questions and the data extraction plan. The reporting of the scoping review was guided by the PRISMA-ScR (9).

### 2.2 Eligibility criteria

The eligibility criteria were guided by the PCC framework (8):

• Population: The review incorporated guidelines pertaining to any demographic, globally or in a specific region or country, that utilizes assistive technology or stands to gain from its use.
• Concept: The focus was on assistive technology in general, specific assistive products, or related services for users or their caregivers. Guidelines including recommendations for these concepts were eligible for the scoping review. Guidelines, focusing on system related recommendations, such as financing models, procurement mechanisms or workforce capacity building were excluded.
• Context: Guidelines containing recommendations for the use of assistive products or related service provision at community or home care, primary care, or specialty care (i.e., advanced, specialized care in specific medical fields) were considered.

This scoping review included guidelines published by international organizations such as the WHO, national or international health or assistive technology associations, professional organizations, governmental bodies (e.g., ministries of health, education, social welfare, defense, etc.), and consortia of health research or academia.

Guidelines either in English or available in English translation and published between January 2008 and March 2024 were eligible for inclusion. Considering the rapid advancements in assistive technology development, such as innovations brought by smartphones, wearables, and artificial intelligence in healthcare, guidelines from the past 15 years should have captured the most relevant and up-to-date recommendations of interest for this review.

### 2.3 Information sources

The sources searched included health science publication outlets with a specific filter on clinical practice guidelines, including PubMed, CINAHL, TRIP, and WHO IRIS. In addition, Google Scholar was searched to complement the aforementioned outlets for any relevant guidelines. The final search strategy and terms were piloted and improved in collaboration with an experienced librarian from the University of Oxford. All searches were conducted in April 2024.

### 2.4 Search

An initial limited search of PubMed and TRIP medical database (10) was executed using broad terms of “assistive technology” or “assistive products” or “assistive devices” and filter “guidelines” to identify records on the topic. The initial search yielded few relevant guidelines. Augmenting the search with terms presenting the functional domains: “cognition”, “communication”, “continence”, “hearing”, “mobility”, “self-care” and “vision” led to more relevant guidelines. Index terms from these relevant guidelines, such as “disability”, “ageing”, “chronic diseases”, “occupational therapy” as well as specific assistive product types identified as having a high prevalence of population need from the most recent surveys (3, 11, 12) were included in the search terms to capture relevant guidelines. In Supplementary Material I, the complete search strategy for each included database is presented.

### 2.5 Selection of sources of evidence

Citations were imported into Zotero 6.0.62 to generate BibTex files, which were subsequently imported to Rayyan (13), an online systematic review support software, for systematic screening. Duplicates were identified by Rayyan using its automatic detection algorithm. Each identified duplicate was examined before removal. Keywords as used in the search terms, such as types of assistive products and functional domains, were highlighted in the title and abstracts by Rayyan to assist the screening. A document was identified as a guideline for this review when the term “*guideline*” was used to describe the work in the title or body of the text.

The reference lists of the included guidelines were reviewed, and potentially relevant citations were retrieved and screened for inclusion by the first author (WZ).

### 2.6 Data charting process

To ensure a structured and comprehensive synthesis of data from the included guidelines, relevant information was extracted and organized using the following methods:

1. A data extraction instrument was developed (see Supplementary Material II), which included the title for the guideline, the organizations leading its development, and the year of publication or the latest update. Content of the guidelines was extracted following the PCC framework (population, concept, context), and outcomes the recommendations intend to achieve. In addition, data extraction also examined stakeholder involvement and methodology followed during the development as well as the presentation of the guideline's recommendations.
2. To verify the extracted data, GPT-4o (https://chatgpt.com/) was used. The verification focused on whether the data extraction had identified all recommendations related to assistive technology. To conduct the verification, the definition of assistive products and the list of 50 priority assistive products (1) were uploaded to GPT-4o first. After that, the full text of the selected guideline was uploaded to GPT-4o to extract the relevant assistive product concept according to the definition and the product list. Identified concepts were compared between manual and automatic extractions. Wherever discrepancies occurred, (re)judgement and a final decision were made by the authors.
3. To present key findings, data addressing the review questions were compiled, summarized narratively, and visualized.

### 2.7 Data items

To systematically chart extracted data in a structured format, a table with 16 columns was developed (see Supplementary Material III).

### 2.8 Synthesis of results

Quantitative data on type of organization, country of organization, year, functional domain, and context are summarized in bar and pie charts. Key themes, trends, and gaps were identified and are narratively described in a structured manner by the review questions.

## 3 Results

### 3.1 Selection and characteristics of sources of evidence

**Figure 1:**
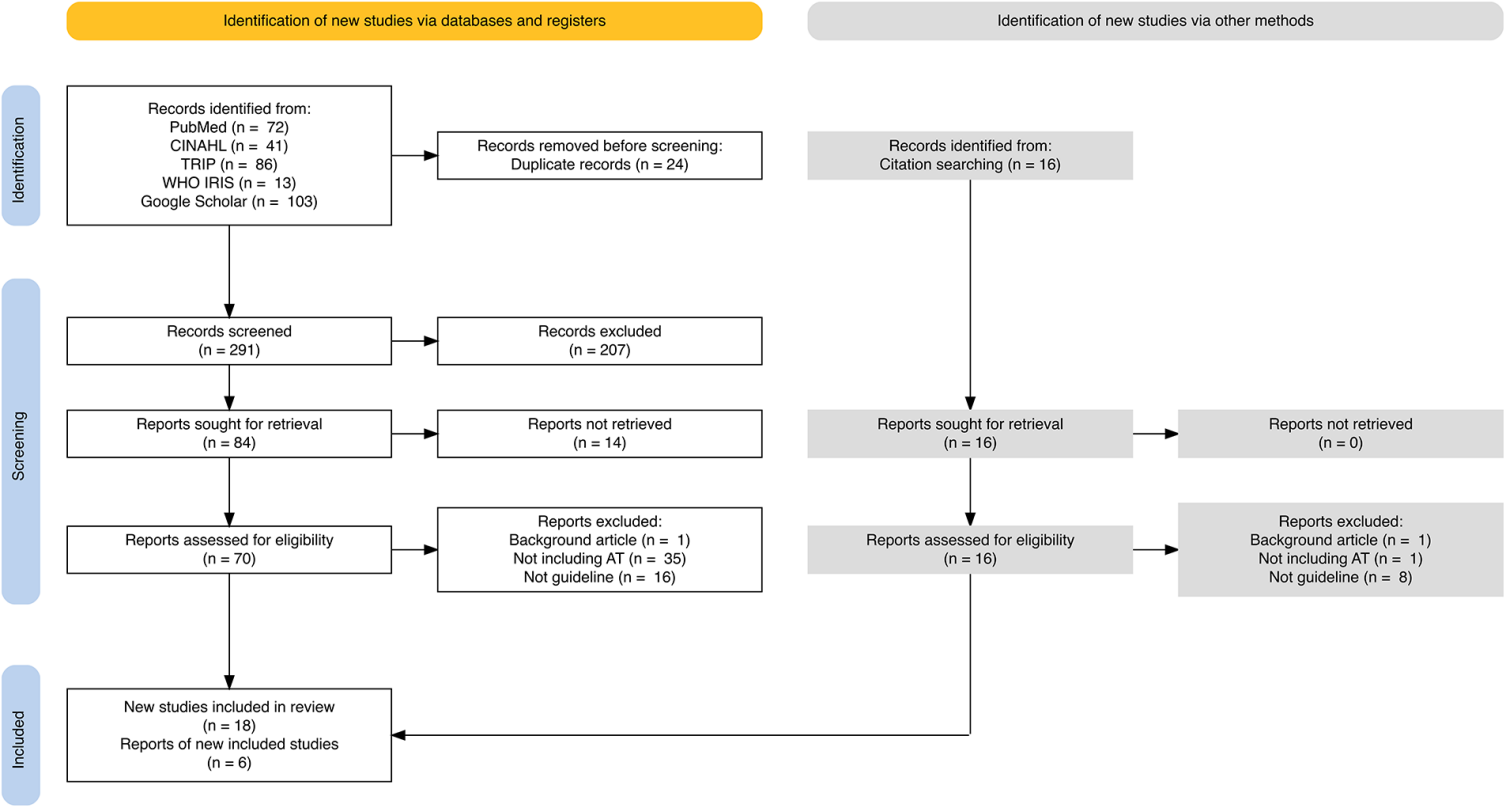
Flow diagram of search results and screening process for the scoping review.

The search returned 72 records from PubMed, 41 from CINAHL, 86 from TRIP, and 13 from WHO IRIS. Records returned in the first 11 pages (10 records per page) in the search of Google Scholar were scanned and 103 records were imported to Rayyan successfully.^2^ The reason to stop scanning after 11 pages was because fewer records' titles were found relevant after that. The search resulted in 315 records in total. After 24 duplicates were removed, 291 records were left for screening. Out of these records, 84 of the records' titles and abstracts were found relevant to the inclusion criteria, and their full documents were sought. Fourteen of these records could not be retrieved in full due to either the full text not being found (*n* = 10) or not being available in English (*n* = 4). Among the 70 full texts screened, 52 were excluded because these documents did not explicitly discuss any assistive product or related services or assistive technology in general (*n* = 35), were background papers (*n* = 1), or were not guidelines (*n* = 16). One guidance statement (14) was included because it was developed specifically based on existing guidelines. Another document (15) was included as it provided comprehensive information from its original guideline published by the Japan Audiological Society, whose full English translation could not be retrieved. Hence, 18 of the 70 full texts were included in the review. While screening the full texts, 16 new records were found relevant through citation searches. After screening the full texts of these additional documents, 6 were added to the review (excluding background papers: *n* = 1, not explicitly discussing assistive technology: *n* = 1, and non-guideline: *n* = 8). Finally, 24 relevant guidelines published in the past 15 years were identified. The full data extraction from the guidelines is presented in Table 1. Figure 1 presents the search results and the screening process through a PRISMA flow diagram for scoping review (9).

**Table 1:** Data extraction of guidelines related to assistive technology.^3^

Title of guidelines (1.1)	Organization (1.2)	Type of organization	Country of organization	Year (1.3)	Population (2.1)	Specific product (2.2)	Specific service (2.2)	AT functional domain	Context (2.3)	Care level	Outcome (2.4)	Target Population Preferences and Views (3.1)	Target Guideline Users (3.2)	Development Approach (4.1)	Recommendation presented clearly (5.1)
Is there an evidence-based efficacy for the use of foot orthotics in knee and hip osteoarthritis? Elaboration of French clinical practice guidelines (28)	SOFMER (French Physical Medicine and Rehabilitation Society)	National professional association	France	2008	Adults with knee and hip osteoarthritis	Foot pronation orthotics, laterally wedge insoles, rearfoot wedge foot orthotics,	Not specific	Mobility	Clinical settings involving management of knee and hip osteoarthritis, focusing on non-pharmacological treatments	Specialty	Effectiveness and the place of FO in the management of knee and hip OA	No specific mention of considering patients’ preference in the development or recommendations	Physicians in Physical Medicine and Rehabilitation, Rheumatologists, Orthopaedic Surgeons	Evidence-based, expert opinion	Yes.
Guidelines on the provision of manual wheelchairs in less-resourced settings (18)	World Health Organization (WHO)	International organization	International	2008	People with disabilities in less-resourced settings, including children, adults, and the elderly	Wheelchair	Design and production, service delivery, policy and planning, user involvement in provision	Mobility	Less-resourced settings, including rural, semi-urban, and urban environments in developing countries	Various	Improved personal mobility, enhanced quality of life, and increased social and economic participation and country system development	Yes. The guidelines emphasise user involvement in design and selection processes to ensure the wheelchairs meet their needs	Government and nongovernmental policymakers, wheelchair service providers, designers, testers, donors, purchasers, adapters of wheelchairs, planners and managers of wheelchair production, developers of training programs, disabled people's organizations, users and their families	Stakeholder consultation, consensus-based	Yes.
Guidelines for the evaluation of hearing aid fitting (15)	Japan Audiological Society	National professional association	Japan	2010	Hearing-impaired individuals	Hearing aids	Evaluation of product efficacy and fitting	Hearing	Clinical practice settings, including general otolaryngology clinics, for fitting and evaluating hearing aids.	Specialty	Improved fitting and efficacy of hearing aids, ensuring they meet the needs of hearing-impaired individuals by improving speech understanding and tolerance to environmental noise.	Yes. The guideline recommended questionnaires assessing subjective hearing aid benefit in daily life scenarios for assessments of fitting outcomes.	Audiologists, otolaryngologists, hearing aid specialists, and other healthcare professionals involved in fitting and managing hearing aids.		Yes.
Community-Based Rehabilitation: CBR Guidelines (19)	World Health Organization (WHO), UNESCO, ILO, International Disability Development Consortium (IDDC)	International organization	International	2010	People with disabilities, including children, adults, and elderly individuals	Spectacles, hearing aids, walking aids.	Not specific	Vision, Hearing, Mobility	Low and middle-income countries, community-level settings	Primary	Improved access to rehabilitation services, enhanced participation and inclusion of people with disabilities in society, and improved quality of life.	Yes. The guidelines emphasise involving disabled people and their families in the development and decision-making processes to ensure their needs and preferences are met.	Primary audience: CBR managers; Secondary audience: CBR personnel, primary health workers, social workers, community development workers, disabled people and their families, DPOs, government officials, development organizations, researchers, and academics.	Principle, field validation	Yes.
Development of clinical guidelines for the prescription of a seated wheelchair or mobility scooter for people with traumatic brain injury or spinal cord injury (23)	Service Development & Review, Lifetime Care & Support Authority, Lukersmith & Associates, Brain Injury Rehabilitation Unit, Liverpool Hospital, Australia	Academic consortia	Australia	2013	Adults with spinal cord injury and/or traumatic brain injury	Seated wheelchairs, mobility scooters	Provision services from assessment to training users on maintenance of devices, and focusing on prescription of the right devices	Mobility	Not specifically mentioned. But likely require specialist capacity	Specialty	To reduce the potential for poor wheelchair prescription.	Yes. Recommendations emphasised people-cantered services where prescription of devices considering the user's anticipated activities in the possible environments.	Occupational therapists	Evidence-based	Yes.
Detection and Nonoperative Management of Paediatric Developmental Dysplasia of the Hip in Infants up to Six Months of Age (35)*	American Academy of Orthopaedic Surgeons (AAOS)	National professional association	USA	2014	Infants up to six months of age with paediatric developmental dysplasia of the hip (DDH)	Wheelchairs, bracing for DDH	Not specific	Mobility	Guidelines applicable in various healthcare settings, involving paediatricians, family physicians, radiologists, and orthopaedic surgeons	Various	Improved detection and management of hip instability and dysplasia in infants, early intervention to prevent progression	Yes. Involvement of patients’ guardians in decision-making, mutual communication between guardians and healthcare providers	Healthcare professionals, including paediatricians, family physicians, radiologists, orthopaedic surgeons, and mid-level practitioners	Evidence-based, consensus-based	Yes.
Management of Falls in Community-Dwelling Older Adults: Clinical Guidance Statement From the Academy of Geriatric Physical Therapy of the American Physical Therapy Association (14)*	Academy of Geriatric Physical Therapy of the American Physical Therapy Association	National professional association	USA	2015	Community-dwelling older adults	Mobility aids, hip protector	User training	Mobility, Self-care	Clinical settings, including physical therapy and multidisciplinary care teams	Various	Improved identification and management of fall risk, reduction in fall rates, better health outcomes	Yes. Inclusion of older people in guideline review. Recommendations emphasised including older people's values and preferences in decision-making, emphasis on tailored interventions	Physical therapists	Evidence-based	Yes.
Guidelines for Adult Stroke Rehabilitation and Recovery (17)*	American Heart Association/American Stroke Association	National professional association	USA	2016	Adults recovering from stroke	Ankle-foot orthosis (AFO), resting ankle splints, electrical stimulation, strapping and taping	Not specific	Mobility	Applicable in various healthcare settings including acute hospital care, inpatient rehabilitation facilities, skilled nursing facilities, home healthcare agencies, and outpatient settings.	Various	Improved stroke rehabilitation outcomes, enhanced recovery, reduced disability, better patient care, and effective organization and coordination among care providers.	Yes. The guideline emphasises patient and family education, patient-cantered care, and inclusion of patient preferences in rehabilitation planning.	Healthcare professionals including physicians, nurses, physical therapists, occupational therapists, speech-language pathologists, psychologists, social workers, and others involved in stroke rehabilitation. The guidelines considered questions interested by policy makers, such as organization and cost.	Evidence-based	Yes.
Suggested Guidelines for the Prescription of Orthotic Services, Device Delivery, Education, and Follow-up Care: A Multidisciplinary White Paper (21)	A task force of experts from universities and clinics in USA, with support from American Orthotic & Prosthetic Association.	Academic consortia	USA	2016	Patients needing orthoses, especially Medicare beneficiaries	Off-the-shelf and custom-fit orthoses	Prescription and delivery of orthotic services	Mobility	Multidisciplinary orthotic care involving orthopaedics, physical medicine physicians, therapists, and certified orthotists.	Specialty	Provide expert guidance for patient safety, minimise wasted expenditures, maximise clinical outcomes, and ensure efficient delivery of care for Medicare and other patients.	No specific mention. It discussed involvement of a multidisciplinary task force including experts in orthopaedics and orthotics to provide patient-centred care. But not specifically recommend if and how to take patients’ preference, values in the care delivery.	Healthcare professionals including physicians, therapists, certified orthotists, and Medicare providers.	Consensus-based	Not in a structured format.
Clinical Practice Guideline (Update): Earwax (Cerumen Impaction) (25)	American Academy of Otolaryngology—Head and Neck Surgery Foundation	National professional association	USA	2017	Individuals >6 months with cerumen impaction	Hearing aids	Detection and user training	Hearing	Any setting where cerumen impaction would be identified, monitored, or managed	Various	Better identify patients with cerumen impaction who may benefit from intervention and to promote evidence-based management to improve management, prevent symptoms, and ensure proper diagnosis	Yes. Recommendation emphasis on considering patient preference wherever evidence for action is doubtful and emphasis on patient education and counselling	All clinicians who are likely to diagnose and manage patients with cerumen impaction.	Evidence-based	Yes.
Osteoporosis prevention, diagnosis and management in postmenopausal women and men over 50 years of age (27)*	The Royal Australian College of General Practitioners and Osteoporosis Australia	National professional association	Australia	2017	Postmenopausal women and men over 50 years of age	Hip protector, bifocal/multi-focal/single strength glasses	Not specific	Self-care, Vision	Community and healthcare settings in Australia	Primary, Home/community	Improved diagnosis, management, and prevention of osteoporosis-related fractures	Yes. The Working Group supports all recommendations and intends that they are used in conjunction with clinical judgement and patient preferences.	Healthcare professionals including general practitioners and specialists	Evidence-based, consensus-based	Yes.
Integrated care for older people: Guidelines on community-level interventions to manage declines in intrinsic capacity (32)*	World Health Organization (WHO)	International organization	International	2017	Older people experiencing declines in intrinsic capacity	Hearing aids, spectacles, alarms, walking aids.	Screening, provision,	Hearing, Vision, Mobility	The interventions are designed to be implemented through models of care that prioritise primary care and community-based care. This includes a focus on home-based interventions, community engagement and a fully integrated referral system.	Primary, Home/community	Improved management of physical and mental capacities, prevention of care dependency, and support for caregivers	Yes. Consideration of values and preferences of older people, inclusion of stakeholders’ views was recommended.	Healthcare providers, program managers, professionals developing training curricula, NGOs, and charities	Evidence-based	Yes.
A Guideline of using Assistive Technologies and Educational Services for Students with Disabilities in Higher Education (20)	Department of Special Education, Faculty of Education, Chiang Mai Rajabhat University, Thailand; Department of Occupational Therapy, Faculty of Associated Medical Sciences, Chiang Mai University, Thailand	Academic consortia	Thailand	2017	Students with disabilities in higher education	Vision, hearing, mobility, cognition, communication products	Not specific	General	Higher education institutions in upper northern Thailand	Others	Accessibility to higher education for students with disabilities	Yes. Views from 12 service providers and 26 undergraduate students with disabilities collected via semi-structured interviews. The guideline also recommends considering values of the students.	Service providers, students with disabilities, educational institutions, governmental and non-governmental organizations	Semi-structured interview	
Clinical Practice Guidelines for the Rehabilitation of Lower Limb Amputation: An Update (24)	US Departments of Defense and Veterans Affairs	Other national authority	USA	2019	Lower limb amputation patients of all ages	Prostheses, rigid or semirigid dressing, microprocessor knee units	Provision of prostheses	Mobility	Not specifically mentioned. Likely require specialist capacity	Specialty	Improved daily function and quality-of-life in patients with LLA	Yes. Patient values and preferences are considered for the guideline development. The guideline provides a framework for managing persons with LLA in the context of their individual needs and preferences.	Healthcare professional working with LLA patients	Evidence-based	Yes.
Risk Reduction of Cognitive Decline and Dementia: WHO Guidelines (29)*	World Health Organization (WHO)	International organization	International	2019	Adults with normal cognition or mild cognitive impairment (MCI)	Hearing aids	Screening	Hearing	Global, applicable to healthcare providers, policymakers, and the general population	Various	Delay or prevent cognitive decline and dementia, improve quality of life and functional level.	Yes. Promotes informed decision-making, considers individual needs and preferences, patient-cantered approach	The guidelines are primarily targeted at health care providers. Quality improvement teams at all levels of the system will benefit from the work. Guidelines and their derivative products have implications for policymakers, health care planners and programme managers at national and international level, as well as the general population.	Evidence-based	Yes.
Urinary Incontinence and Pelvic Organ Prolapse in Women: Management (16)*	National Institute for Health and Care Excellence (NICE)	National health authority	UK	2019	Women aged 18 and over with urinary incontinence, pelvic organ prolapse, or complications associated with surgery for these conditions.	Absorbent products, hand-held urinals, and toileting aids, vaginal pessaries.	Regular assessment and proper fitting	Self-care, continence	Applicable in various healthcare settings, including primary care and specialist services. It also addresses multidisciplinary team involvement and regional MDTs for complex cases.	Primary, Specialty	Improved management and treatment of urinary incontinence and pelvic organ prolapse, reduction in symptoms, better patient care, and management of complications related to mesh surgery.	Yes. Emphasises involving patients in decision-making processes, considering individual needs, preferences, and values, and promoting informed decisions about their care.	Healthcare professionals, service commissioners, and women with urinary incontinence, pelvic organ prolapse, or related surgical complications, including their families and carers.	Evidence-based	Yes.
Occupational Therapy Practice Guidelines for Early Childhood: Birth until 5 Years (34)*	American Occupational Therapy Association (AOTA)	National professional association	USA	2020	Children ages birth until 5 years	Wetting alarm	Not specific	Continence	Various early childhood settings, including home, community, hospital, and educational environments	Primary, Home/community, Others	Effectiveness of interventions to support the development of cognitive, social–emotional, motor, and self-care skills	Yes. People-cantered care with a focus on family involvement; interventions tailored to individual needs and preferences	Occupational therapists and occupational therapy assistants, as well as the people who manage, reimburse, or set policy regarding occupational therapy services. Can also be a reference for broad audience such as researchers and carers.	Evidence-based	Yes.
Occupational Therapy Practice Guidelines for Older Adults With Low Vision (33)	American Occupational Therapy Association (AOTA)	National professional association	USA	2020	Older adults with low vision	Magnifiers (CCTV, OrCam, low vision devices)	Not specific	Vision	Various healthcare settings, focusing on rehabilitation	Various	Improved service delivery, and quality of care, enhanced consumer satisfaction, and justified occupational therapy services to external stakeholders	Yes. Recommend using client-cantered problem-solving training to enhance ADL and IADL performance, reading, and leisure and social participation	Occupational therapists and occupational therapy assistants, as well as the people who manage, reimburse, and set policy regarding occupational therapy services. This guideline can also serve as a reference for health professional, regulators, and researchers.	Evidence-based, expert opinion	Yes.
Automated seizure detection using wearable devices: A clinical practice guideline of the International League Against Epilepsy and the International Federation of Clinical Neurophysiology (26)	International League Against Epilepsy and the International Federation of Clinical Neurophysiology	International professional association	International	2021	Children and adults with epilepsy, who are not seizure-free and who have either (1) GTCS, including FBTCS or (2) focal impaired awareness seizures, without tonic-conic component.	Room/bed-placed sensor, wearable devices/alarms	Not specific	Self-care, environment-modification	Outpatients with epilepsy in ambulatory settings	Home/community	Sensitivity, false alarm rate, adverse events, usability of the interventions to decrease morbidity and mortality associated with seizures and for objective seizure identification and quantification.	Yes. The guideline emphasises costs, patientś preferences and perspectives should be considered in the evaluation of impact of this technology.	Healthcare personnel working with patients with epilepsy	Evidence-based	Yes.
Guidelines of the French Society of Otorhinolaryngology-Head and Neck Surgery (SFORL) and the French Society of Audiology (SFA) for Speech-in-Noise Testing in Adults (30)	French Society of Otorhinolaryngology-Head and Neck Surgery (SFORL) and the French Society of Audiology (SFA)	National professional association	France	2022	Adults with hearing loss, specifically those undergoing speech-in-noise testing	Hearing aids, bone-anchored bone-conduction devices.	Test for hearing gain/efficacy	Hearing	Various healthcare settings, including clinical and research settings	Specialty	Improved assessment and management of speech intelligibility in noise for adults with hearing loss.	No specific mention of considering users’ preference in the development or recommendations.	ENT physicians, audiologists, audio prosthetists, and other healthcare professionals involved in hearing care	Evidence-based and expert opinion	Yes.
World guidelines for falls prevention and management for older adults: a global initiative (39)*	British Geriatrics Society	National professional association	UK	2022	Older adults aged 65 and over	Wearables, personal emergency alarms, handrail/grab bar	Not specific	Mobility, Environment- modification	Global initiative, applicable in community, care homes, and hospitals	Various	Reduction in falls, improved functional mobility, reduced fall-related injuries	Yes. Inclusion of older adults’ perspectives, caregivers' views in the guideline development and emphasised the including older people's beliefs, attitudes and priorities in management of falls.	Healthcare professionals including physicians, nurses, physiotherapists, occupational therapists, pharmacists, and allied health professionals	Consensus-based	Yes.
Management of Upper Limb Amputation Rehabilitation (31)	US Department of Veterans Affairs and US Department of Defence	Other national authority	USA	2022	Adults (≥18 years) with acquired upper limb amputation, including veterans as well as service members, military retirees, and beneficiaries.	Upper limb prostheses	Product design and fitting	Mobility	The updated guideline in 2022 includes an additional treatment algorithm that is designed specifically for primary care providers	Primary, Specialty	Improved quality of life in patients with ULA	Yes. Patient values and preferences are taken into account for the guideline development where it utilised gender-specific patient focus groups to identify priority clinical issues and gender-specific management considerations.	Healthcare providers engaged in the care of patients with ULA	Evidence-based	Yes.
Wheelchair provision guidelines (22)	World Health Organization (WHO)	International organization	International	2023	Children, older persons, people with mobility disabilities, and those with chronic health conditions	Wheelchair	Individualised assessment, fitting and preparation, integrated services, systematic evaluation, and user training,	Mobility	Healthcare settings, including primary, secondary, and tertiary health facilities, and outreach to community settings	Various	Improved access to appropriate wheelchairs, ensuring mobility, inclusion, and participation at both individual service and system levels	Yes. Involvement of wheelchair users in guideline development and in decision-making processes, emphasis on user engagement and choice in the recommendation	Policymakers, healthcare providers, wheelchair service personnel, and representatives of wheelchair users	Evidence-based, consensus-based	Yes.
WHO guideline for non-surgical management of chronic primary low back pain in adults in primary and community care settings (13)*	World Health Organization (WHO)	International organization	International	2023	Adults with chronic primary low back pain	Wheelchairs, mobility scooters, crutches, walking sticks/canes, and walking frames/walkers, lumbar braces belts and supports	Not specific	Mobility	Non-surgical interventions can be delivered in primary and community care settings	Primary, Home/community	Improved health and well-being outcomes related to CPLBP	Yes. The values and preferences of people with CPLBP and their families and health workers relating to the interventions and their outcomes as well as the acceptability and feasibility of the interventions were considered.	Health workers of all disciplines working in the primary and community care settings, and discipline neutral	Evidence-based	Yes.

Records with * are verified by GPT-4o for data extraction.

Data extraction was verified by GPT-4o on 10 records (42% of the total reviewed records). These guidelines are oriented around health condition (such as falls, stroke, osteoporosis, urinary incontinence, etc.) instead of assistive technology, and the conditions likely concerned multiple assistive product interventions. Among the verified records, discrepancies were found in two records. GPT-4o identified vaginal pessaries (16) and electrical stimulator (17), which were not identified by the author. These two products fall under the definition of assistive products and the ISO 9999 classification. Hence, they were included in the data extraction.

**Figure 2:**
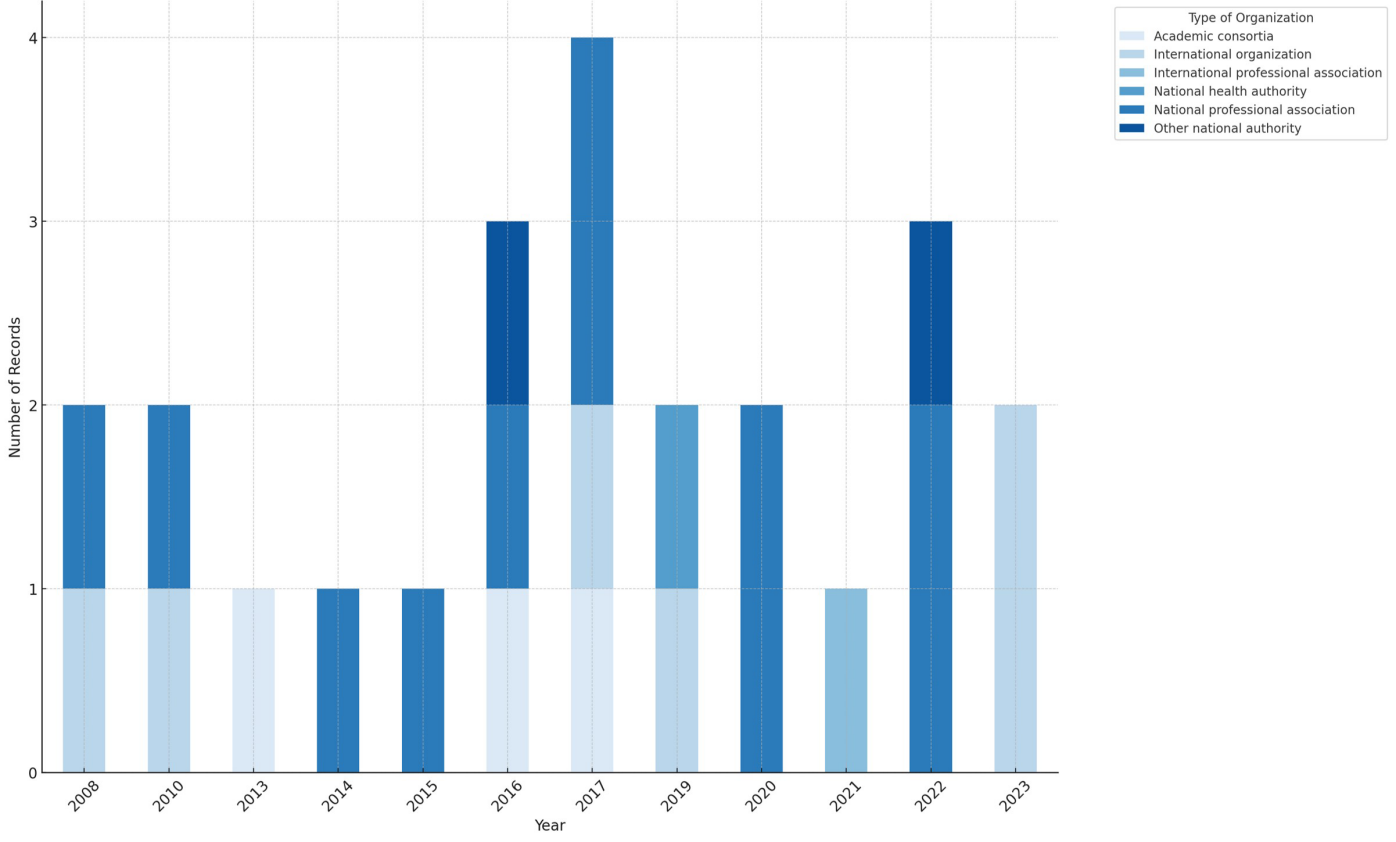
Identified guidelines by year, country, and type of developing organization.

**Figure 3:**
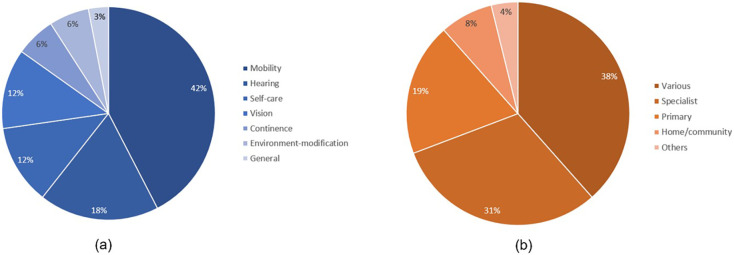
Percentages of assistive technology functional domains **(a)** and percentages of care context **(b)** presented in the guidelines.

Figure 2 illustrates the general demographic information of these guidelines, including the year of publication, the countries, and the types of developing organizations. Figure 3 summarizes the distributions of functional domains and care settings addressed by the identified guidelines related to assistive technology. Findings responding to the review questions on the availability of guidelines are summarized in the following subsections, with discussions deepening into the types of products and services covered, populations and typical health conditions addressed, applicable healthcare settings for interventions, as well as the methodologies used for the development of these guidelines.

### 3.2 Availability of guidelines related to assistive technology

National health professional organizations, predominantly from high- and upper-middle-income countries [USA (*n* = 9), UK (*n* = 2), France (*n* = 2), Australia (*n* = 2), Japan (*n* = 1) and Thailand (*n* = 1)], have been instrumental in producing guidelines for assistive technology. Of the 24 identified guidelines, 11 were created by professional associations in fields like audiology, geriatrics, physical medicine, and rehabilitation. These guidelines, including contributions from the WHO, which collaborated with international professional associations, aim to establish high standards for screening, assessment, and intervention in assistive technology. The primary aim of these guidelines is to improve health outcomes by providing systematic methods for selecting and utilizing assistive products, though some also address system-level outcomes, including the enhancement of national service provision and regulation of product standards.

Guidelines published by international professional association (*n* = 1) and WHO (*n* = 6), generally developed in collaboration with other agencies, tend to have an international scope, with some (*n* = 2) addressing specific needs in low-resource or low-income settings (18, 19). Guidelines developed by national organizations often target the unique needs of their country's population, though some are adapted for broader applicability.

### 3.3 Assistive products and related services covered

Guidelines predominantly cover mobility products (*n* = 14), which include wheelchairs, walking aids, orthoses, and prostheses. This is followed by those for hearing (*n* = 6), self-care (*n* = 4), vision (*n* = 4), continence (*n* = 2), and environment-modification (*n* = 2). One guideline recommends assistive technology in general (20). Cognitive and communication products are notably absent, indicating a gap in assistive technology guidelines for these functions. Eight guidelines include recommendations of assistive products for more than one functional domain.

Most guidelines, especially those focusing on mobility and hearing products, emphasize the importance of individualized assessments to ensure that devices are matched to user needs. These assessments are crucial in adjusting products to fit the users and providing training to optimize the products' benefits. Some guidelines provide detailed protocols for managing the provision of specific assistive products, such as wheelchairs or orthoses, which include steps for design, production, qualification of the workforce, and policy considerations (18, 21–24). Guidelines on wheelchair provision (22), for example, outline proper fitting procedures, recommended materials, and user training to maximize mobility benefits. Less specificity is found in guidelines where assistive technology is one element of a broader intervention package, such as those addressing self-care, incontinence, or environmental accessibility improvements (25, 26). Majority (21 of 24) guidelines recommend that users' preferences and values be considered during the selection and delivery of assistive products.

### 3.4 Populations addressed

Several identified guidelines specifically address adults living with chronic health conditions or disabilities, such as those with stroke (17), osteoarthritis (27, 28), cognitive impairment (29), hearing loss (30), chronic primary low back pain (31) and acquired upper limb amputations (32). Older adults are often a key demographic, with guidelines targeting them for assistive products like mobility aids (14, 33) and fall prevention measures (34). According to one guideline (27), older people should not be prescribed bifocal or multifocal spectacles for outdoor use due to an increased risk of falls. Two guidelines address the assistive technology applications for children, focusing on early childhood development (35) and managing conditions like developmental dysplasia of the hip (36). Female adults are the target population in two guidelines focused on osteoporosis (27) and urinary incontinence management (16). Students with disabilities are the focused demographic in one guideline, which recommends assistive technologies that facilitate access to education and navigation within school environments (20).

### 3.5 Applicable contexts

The identified guidelines address various healthcare settings, including specialty care (*n* = 8), primary care (*n* = 7) and home/community care (*n* = 5). The settings vary depending on the complexity of the assistive product and the severity of the user's health condition. Specialty care is mainly recommended for complex products like prostheses and hearing aids that require individualized fitting and adjustments by professionals. Primary care settings address simpler assistive products such as mobility aids, absorbent products, and alarms for emergency response, making them accessible within a community context. Guidelines suggest home and community care services are suitable for providing simple aids, like vision or mobility products, which enable easier management of daily activities. Besides healthcare settings, the education (school) environment is included in two guidelines for assistive technology provision to support learning and accessibility.

### 3.6 Development methods

The majority (*n* = 18) of the identified guidelines were developed using evidence-based methodologies that include literature reviews, evidence synthesis, and stakeholder consultations, as specified in their respective institutional development guidance. For example, the WHO handbook for guideline development (7), is the guidance framework with oversight by an organizational review committee. The framework adopts standardized evidence appraisal framework, such as GRADE (37) and AGREE II (38) to ensure methodological rigor, as several professional associations do for their guideline development. Aiming for transparency, most guidelines present recommendations based on evidence quality. However, the evidence to support recommendations for assistive products was generally weak in the reviewed guidelines. Other development methodologies include consultative and consensus-based approach (18, 21, 39), or based on principles and field validations (19), or using a qualitative research approach such as semi-structured interviews (20). Patients and caregivers' involvement in the development were discussed in some guidelines.

## 4 Discussions

The findings underscore assistive technology's role in managing a diverse range of health conditions across all age groups. Guidelines aimed at condition-specific interventions address assistive technology within broader treatment recommendations, reflecting its integrative role in health management. The discussion of considering users' preference in product selection and delivery by many guidelines emphasizes user-centered provision model for assistive technology.

However, the review reveals key limitations in the global landscape of assistive technology guidelines, where focus is on products for vision, hearing, mobility, and self-care. There are significant gaps in guidelines for products for cognition and communication, as well as assistive technology-specific service provision for most product categories. While evidence-based approaches are widely adopted, limitations in high-quality research on assistive technology and patients' and caregivers' involvement in guideline development process can affect the strength and the applicability of recommendations.

These findings highlight a need for more diverse guideline development for improving access to assistive technology, especially in low-resource settings. As assistive technology plays an increasing role across healthcare, especially for aging populations and those living with chronic health conditions, efforts to strengthen guidelines can contribute to more accessible, inclusive healthcare. The following discussion addresses major challenges impacting assistive technology guideline development and adoption into policies and practices. Opportunities and potential actions for improving future assistive technology research and guideline development are outlined.

### 4.1 Challenges in assistive technology policy and practices for health interventions

#### 4.1.1 Ambiguity in international standards for assistive products

Inconsistency in classifications and terminologies for assistive products among international standards is most notable between the ISO 9999 and the WHO APL—the two most prominently referenced standards in the field. Challenges also arises due to incomplete information as neither abovementioned list provides clear descriptions of the included products. Lack of clarity and inconsistency create ambiguity and makes communication of assistive technology concepts challenging among stakeholders (40). From manufacturing, procurement, and trade to systematic evidence generation of products' safety and efficacy profiles, adopting unified international terminologies and standards is critical.

Additionally, the rapid evolution of digital technology, such as smartphones integrating assistive functions, challenges existing standards. For example, categories like video communication devices are now outdated, as many of its functions are integrated into general consumer electronic devices, and no longer exclusively used by people with disabilities. This makes the concept of assistive technology diverse beyond disability and will continue evolving (41). These discrepancies highlight the need for adaptable, future-proof terminologies and standards that can accommodate both traditional and emerging assistive products.

#### 4.1.2 Lack of evidence-informed provision

A recent scoping review specifically on guidelines for assistive products provision had found that this topic being discussed mostly in book chapters, scientific articles or proceedings, and reports (42). The development of reliable recommendations for provision of assistive products is hampered by limited high-quality evidence (43) and the absence of standardized outcome measures (44), even in some of the most developed areas like wheelchairs (22). Lack of evidence-informed provision could be responsible for the observation of beneficiaries receiving inappropriate or unusable assistive products due to poor service provision (45). Assistive technology interventions are often complex, involving multiple products tailored to individual needs, which complicates evidence-generation based on randomized controlled trials (43).

Without consistent data, assistive technology providers struggle to make informed decisions, leading to suboptimal product selections that may not fully benefit users. In the absence of robust evidence, guideline recommendations are often broad, rather than tailored to specific products and user profiles, impacting the outcome of the intervention.

#### 4.1.3 Inadequacy of professional workforce

Effective assistive technology provision requires specialized skills across multiple domains, yet training opportunities are limited (3, 46). Few well-established assistive technology professions, such as audiologists, opticians, orthotists, and prosthetists, focus on specific areas of assistive technology without extensive cross-functional collaboration. The shortage and siloed professions slow the advancement of cross-functional expertise and weaken the advocacy for policy and practices improvement in the field. As professional groups are a driving force for guideline development, lack of assistive technology professionals and less integrated knowledge in other health professions have left the assistive technology guidelines behind.

#### 4.1.4 Complexity in leadership and governance for policy implementation

Implementing comprehensive assistive technology policies requires cooperation across sectors (47), which can be challenging due to fragmented governance systems, varying budget priorities, administrative and logistical constraints. Guideline implementation can also be hindered by local context, such as constraints in local and global supply chains of assistive products that limiting selection to what available on the local market than population needs. Similar to the constraints have been seen in medicines and vaccines access challenges (48). Furthermore, insufficient public financing or lack of alternative financing models for assistive technology could prevent full service provision as recommended (49). These challenges due to system complexity may demotivate leadership and inefficient governance in prosperity of assistive technology policy and practice implementation, which reversely hinder guideline development and adoption.

### 4.2 Opportunities for improving assistive technology guidelines and practices

#### 4.2.1 Harmonizing norms and standards

Aligning ISO 9999, WHO's APL, and other relevant international standards, such as the International Classification of Functioning, Disability and Health (ICF), which sets international norms on risk factors used by research and clinical practices in physical medicines, rehabilitation, disability studies (50), would improve clarity and streamline assistive technology provision by establishing a consistent framework. Efforts to provide product specifications (51) and training for service provision (52) have laid groundwork for better alignment. Similarly, harmonizing outcome measures is essential for improving the quality and comparability of assistive technology impact assessments (53). Standardizing outcome measures would allow researchers and policymakers to evaluate and compare efficacy and effectiveness of different assistive products and with other health interventions (44, 54, 55) focusing on what matters (56, 57), and providing a stronger evidence base for future guidelines.

It is worth noting that harmonization of norms and standards should be continuous consultative and collaborative processes, aiming towards a common understanding and benefit from relevant and emerging concepts in health outcome measures (58), validated methods and data collection tools (59–64) to enable synergized information on assistive technology.

#### 4.2.2 Identifying and filling evidence gaps through systematic research

Recent initiatives to review the need, benefit, risk and cost for assistive products offer pathways to enhance evidence base for resource prioritization by focusing on cost-effectiveness (65, 66). Studies on modelling the selection of assistive products (67, 68), and barriers and facilitators for delivering interventions (69–71) are critical to advance evidence-informed provision to improve user-centered outcomes. Addressing evidence gaps will require diverse research methodologies, including observational studies and qualitative assessments, to capture real-world usage and its impact on daily life. Such evidence would enable guidelines to provide more specific, actionable recommendations for assistive product provision.

Initiatives on innovative and fit-for-purpose research methodologies could be another cornerstone to advance clinical practices in assistive technology. Opportunities for health research are to be taken to expand from traditional lab-bound and centralized research environment to versatile contexts and decentralized manner. Boosting research and evidence generation with advanced data availability and growing analytical power could derive useful recommendations in the absence of RCTs.

#### 4.2.3 Advancing practice with living guidelines

Implementing a “living guidelines” approach (72), where recommendations are continuously updated with emerging evidence, could help bridge gaps in assistive technology guidance. The WHO has successfully used this approach for COVID-19 guidelines (73) and other areas of work (74), showing its potential for fast-evolving fields like assistive technology. Living guidelines would allow recommendations to evolve in line with technological advancements, such as AI-enabled assistive products, ensuring that practitioners always have access to current, evidence-based guidance. Regular updates would benefit practitioners and policymakers alike, facilitating timely decision-making and better health outcomes for users.

#### 4.2.4 Raising awareness

Initiatives such as the World Day for Assistive Technology are crucial for building awareness about assistive technology needs, especially in low- and middle-income countries (75). By highlighting unmet needs, these campaigns can help mobilize resources and advocate for stronger policies. Increased awareness can also engage communities, policymakers, and healthcare providers, reinforcing the importance of access as a fundamental healthcare service and enhancing funding opportunities. Such campaigns are opportunities to bring all relevant stakeholders together to highlight the multi-sectoral efforts needed for improving assistive technology access for health, education, and social welfare. Adopting a system thinking for policy and practices, as well as the guideline development at its start, is key to delivering interventions to make positive impact on assistive technology users (76).

### 4.3 Actions for stakeholders

Including assistive technology in universal health coverage plays an essential role in the implementation of United Nations Convention on the Rights of Persons with Disabilities and achieving the Sustainable Development Goals (3, 77).

In response to WHA71.8's call, effective translation of research outcomes to policy and practices needs a joint effort of pushing evidence to practices and pulling research to address policy gaps. Assistive technology guidelines development is a key action in this intersection. Following actions are proposed to be considered by the WHO, researchers and practitioners, policymakers (such as ministries, regulators, research ethicists), and other stakeholders (such as global health and development donors, civil societies, and industry).

Researchers and practitioners are in the driving seat of developing innovative research methodology and expansion of evidence generation capturing the full scope of assistive technology impact. This will lead to improvements in provision by focusing on user-centered outcomes. For policymakers, supporting evidence-informed guidelines and policies that prioritize collaboration and user engagement can improve access (76). A coordinated, inclusive policy approach will help address gaps in provision across different sectors. Funders for research and development in assistive technology may consider enhancing resources for knowledge translation in their funding schemes, and enabling researchers' engagement with civil societies, industry, and policy makers to increase the chance of research outcomes being taken up by policy and practice. As a leading agency in global health norm and standard, WHO, with its Global Cooperation on Assistive Technology platform (78), is well-positioned to coordinate international efforts. Continued work on harmonizing and standardizing assistive technology guidelines and promoting evidence-based practices will be instrumental in expanding accessibility.

### 4.4 Strengths and limitations

The strengths of this review include: (1) the inclusion of a diverse range of international and national guidelines provides a broad perspective on current assistive technology practices; (2) the identification of key gaps in evidence and challenges in assistive technology guidelines; and (3) comprehensive discussions of opportunities and actions for stakeholders in future policy development and research aimed at improving evidence-informed guidelines and practices.

This review is limited by language constraints, as it only includes English-language guidelines, potentially excluding relevant local guidelines in other languages. Furthermore, it focuses on specific databases and sources, which may result in the omission of guidelines indexed in other policy document databases such as Overton (https://www.overton.io).

Future studies could consider extending the review by including guidelines in other languages than English, in more databases and grey literatures, as well as expanding search terms such as “adaptive equipment”, “accessibility equipment”, “support devices” to identify relevant guidelines, especially in underrepresented functional domains in this review.

## 5 Conclusions

This scoping review provides a critical overview of the current landscape of assistive technology guidelines. The findings underscore the need to expand and accelerate research and practices in the field to ensure that guidelines are grounded in robust evidence and remain relevant and effective. Furthermore, there is an urgent need for harmonized terminology and international standards to enhance the clarity, consistency and global applicability of assistive technology guidelines.

To advance assistive technology policy and practices, the review highlights the importance of continuous, coordinated, and collaborative efforts that value the perspectives of users and stakeholders in both evidence generation and guideline development. By addressing identified gaps and leveraging opportunities, stakeholders can improve the availability and quality of assistive technology guidelines, ultimately enhancing the health and well-being of people who need assistive technology worldwide.

## Data Availability

The original contributions presented in the study are included in the article/Supplementary Material, further inquiries can be directed to the corresponding authors.

## Footnotes

^1^Due to technical issues, the update was registered in OSF on 6 August 2024.

^2^Unsuccessful importation was due to invalid file links.

^3^Records with * are verified by GPT-4o for data extraction.
