# Supplementary material for: Global availability of guidelines related to assistive technology: a scoping review
Source: Front Rehabil Sci. 2025 Apr 24;6:1581104. doi: 10.3389/fresc.2025.1581104 (PMC12058544; doi:10.3389/fresc.2025.1581104)
Supplement: Supplementary file 1 [file Table1.docx]

Supplementary Material

# Search terms applied in source databases

## Search terms applied in PubMed

(technolog*[Text Word] OR assistive technolog*[Text Word] OR assistive product*[Text Word] OR assistive device*[Text Word] OR spectacle*[Text Word] OR eye glass*[Text Word] OR walking aid*[Text Word] OR walking device*[Text Word] OR walking stick*[Text Word] OR walking cane*[Text Word] OR mobility aid*[Text Word] OR mobility device*[Text Word] OR tripod*[Text Word] OR quadripod*[Text Word] OR crutch*[Text Word] OR shower chair*[Text Word] OR toilet chair*[Text Word] OR bath chair*[Text Word] OR wheelchair*[Text Word] wheel chair*[Text Word] OR hearing aid*[Text Word] OR hearing device*[Text Word] OR orthos*[Text Word] OR prosthe*[Text Word] OR continence aid*[Text Word] OR continence pad*[Text Word] OR incontinence aid*[Text Word] OR incontinence pad*[Text Word] OR "mobile health"[Text Word] OR "mobile app*"[Text Word] OR mhealth[Text Word] OR apps[Text Word] OR app[Text Word] OR digital technolog*[Text Word] OR digital product*[Text Word] OR digital device*[Text Word] OR wearables[Text Word] OR wearable technolog*[Text Word] OR wearable product*[Text Word] OR wearable device*[Text Word] OR smarthome*[Text Word] OR smart home*[Text Word] OR occupational therap*[Text Word] OR ((home*[Text Word] OR shower*[text Word] OR bath*[Text Word] OR toilet[Text Word])AND adapt*[Text Word]))

AND

(disabilit*[Text Word] OR disabled[Text Word] OR "old people"[Text Word] OR "older people"[Text Word] OR old person*[Text Word] OR older person*[Text Word] OR elderly[Text Word] OR "old age"[Text Word] OR aged[Text Word] OR aging[Text Word] OR ageing[Text Word] OR geriatric*[Text Word] OR veteran*[Text Word] OR chronic disease*[Text Word] OR chronic condition*[Text Word] OR long term condition[Text Word] OR "long-term care"[Text Word] OR vision impairment*[Text Word] OR "visually impaired"[Text Word] OR "low vision"[Text Word] OR blind*[Text Word] OR mobility[Title] OR mobility impairment*[Text Word] OR mobility difficult*[Text Word] OR hearing impairment*[Text Word] OR "hard of hearing"[Text Word] OR deaf[Text Word] OR hearing difficult*[Text Word] OR hearing disorder*[Text Word] OR communication[Text Word] OR speaking[Text Word] OR speech[Text Word] OR aphasia[Text Word] OR "cognitive decline"[Text Word] OR cognition impairment*[Text Word] OR incontinence[Text Word] OR continence[Text Word] OR selfcare[Text Word] OR self-care[Text Word])

## Search terms applied in CINAHL

((TI technolog*) OR "assistive technolog*" OR "assistive product*" OR "assistive device*" OR spectacle* OR "eye glass*" OR "walking aid*" OR "walking device*" OR "walking stick*" OR "walking cane*" OR "mobility aid*" OR "mobility device*" OR tripod* OR quadripod* OR crutch* OR "shower chair*" OR "toilet chair*" OR "bath chair*" OR "wheelchair* wheel chair*" OR "hearing aid*" OR "hearing device*" OR orthos* OR prosthe* OR "continence aid*" OR "continence pad*" OR "incontinence aid*" OR "incontinence pad*" OR "mobile health" OR "mobile app*" OR mhealth OR apps OR app OR "digital technolog*" OR "digital product*" OR "digital device*" OR wearables OR "wearable technolog*" OR "wearable product*" OR "wearable device*" OR smarthome* OR "smart home*" OR "occupational therap*" OR ((home* OR shower* OR bath* OR toilet) "AND adapt*")) AND (disabilit* OR disabled OR "old people" OR "older people" OR "old person*" OR "older person*" OR elderly OR "old age" OR aged OR aging OR ageing OR geriatric*" OR veteran* OR "chronic disease*" OR "chronic condition*" OR "long term condition" OR "long-term care" OR "vision impairment*" OR "visually impaired" OR "low vision" OR blind* OR (TI mobility) OR "mobility impairment*" OR "mobility difficult*" OR "hearing impairment*" OR "hard of hearing" OR deaf OR "hearing difficult*" OR "hearing disorder*" OR communication OR speaking OR speech OR aphasia OR "cognitive decline" OR "cognition impairment*" OR incontinence OR continence OR selfcare OR self-care)

## Search terms applied in TRIP

((title:disabilit* OR title:disabled OR title:old OR title:older OR title:elderly OR title:aged OR title:geriatric* title:ageing OR title:aging OR title:veteran* OR title:chronic OR title:"long title:term" OR title:vision OR title:visually OR title:blind* OR title:mobility OR title:hearing OR title:deaf OR title:communication OR title:speaking OR title:speech OR title:aphasia OR title:cognitive OR title:cognition OR title:incontinence OR title:continence OR title:selfcare OR title:self-care) AND ("assistive technolog*" OR "assistive product*" OR "assistive device*" OR spectacle* OR "eye glass*" OR "walking aid*" OR "walking device*" OR "walking stick*" OR "walking cane*" OR "mobility aid*" OR "mobility device*" OR tripod* OR quadripod* OR crutch* OR "shower chair*" OR "toilet chair*" OR "bath chair*" OR "wheelchair* wheel chair*" OR "hearing aid*" OR "hearing device*" OR orthos* OR prosthe* OR "continence aid*" OR "continence pad*" OR "incontinence aid*" OR "incontinence pad*" OR "mobile health" OR "mobile app*" OR mhealth OR apps OR app OR "digital technolog*" OR "digital product*" OR "digital device*" OR wearables OR "wearable technolog*" OR "wearable product*" OR "wearable device*" OR smarthome* OR "smart home*" OR "occupational therap*"))

## Search terms applied in WHO IRIS

Subject equals ‘guideline’ AND contains (‘disability’ OR ‘rehabilitation’ OR ‘aging’ OR 'chronic’ OR ‘mobility’ OR ‘vision’ OR ‘hearing’ OR 'communication’ OR’ self-care’ OR ‘cognition’) AND Language equals ‘English’ AND Date issued equals [2008 TO 2024].

## Search terms applied in Google Scholar

guideline AND assistive AND (technology OR technologies OR product OR products OR device OR devices)
