# Supplementary material for: Global availability of guidelines related to assistive technology: a scoping review
Source: Front Rehabil Sci. 2025 Apr 24;6:1581104. doi: 10.3389/fresc.2025.1581104 (PMC12058544; doi:10.3389/fresc.2025.1581104)
Supplement: Supplementary file 2 [file Table2.docx]

Supplementary Material

# Data extraction instrument

| **ID** | **Index** | **Item** | **Description** |
| --- | --- | --- | --- |
| Part 1: administrative information | | | |
|  | 1.1 | Title | Full title of the guideline and the version, if the guideline has been updated. |
|  | 1.2 | Organization | The organization leading the development of the guideline. |
|  | 1.3 | Year | The latest update of the guideline. |
| Part 2: scope and purpose | | | |
| 1 | 2.1 | Population | Target population described by their demographic attributes (such as age, sex, country or region) and / or the health conditions. |
| 2 | 2.2 | Concept | Specific product types addressed and / or related services (such as referral, need assessment, product design/fitting/adaptation/maintenance, user training). |
| 3 | 2.3 | Context | The environment (such as home/community, primary or higher- level healthcare), where the recommendation is applicable. |
| 4 | 2.4 | Outcome | The health or other outcomes the recommendations intend to achieve (such as improved function, increased participation, etc). |
| Part 3: stakeholder involvement | | | |
| 5 | 3.1 | Target population preferences and view | Methods used to include assistive technology users’ view in the guideline development. |
| 6 | 3.2 | Target guideline users | List of guideline users. |
| Part 4: rigor of development | | | |
| 7 | 4.1 | Methodology | Is the guideline developed following an established methodology? |
| Part 5: clarity of presentation | | | |
| 8 | 5.1 | Recommendations are identifiable | Are the recommendations easily identified in the guideline? |
