# Supplementary material for: Global availability of guidelines related to assistive technology: a scoping review
Source: Front Rehabil Sci. 2025 Apr 24;6:1581104. doi: 10.3389/fresc.2025.1581104 (PMC12058544; doi:10.3389/fresc.2025.1581104)
Supplement: Supplementary file 3 [file Table3.docx]

Supplementary Material

# 3. Data chart items and descriptions.

| **Id** | **Item** | **Description** |
| --- | --- | --- |
| 1 | Title | Full title of the guideline and the version. |
| 2 | Organization | The organization leading the development of the guideline. |
| 3 | Type of organization | The type of organization leading the development of the guidance. |
| 4 | Country | The country of the organization leading the development of the guideline. |
| 5 | Year | Year of the latest update of the guideline. |
| 6 | Population | Target population described by their demographic attributes or the health conditions. |
| 7 | Specific product | Specific product type addressed. |
| 8 | Specific service | Specific service related to assistive products. |
| 9 | Functional domain | Functional domains assistive products addressed (cognition, communication, continence, environment-modification, hearing, mobility, self-care, and vision). |
| 10 | Context | Socioeconomic environment and healthcare settings (e.g., low-resource settings), where the recommendation is intended. |
| 11 | Care level | Level of care for which the guideline is intended (specialty, primary, home/community care, various (or not specifically defined), and others). |
| 12 | Outcome | Health or other outcomes the recommendations intend to achieve |
| 13 | Target population preferences and view | Methods used to include assistive technology users’ view in the guideline development. |
| 14 | Target guideline users | Intended guideline users. |
| 15 | Development approach | Methodology used when developing the guideline (evidence-based, consensus-based, and other approaches) |
| 16 | Recommendations presented clearly | Whether recommendations are easily identified in the guideline. |
