# Supplementary material for: Global availability of guidelines related to assistive technology: a scoping review
Source: Front Rehabil Sci. 2025 Apr 24;6:1581104. doi: 10.3389/fresc.2025.1581104 (PMC12058544; doi:10.3389/fresc.2025.1581104)
Supplement: Supplementary file 4 [file Table4.docx]

Supplementary Material

# Data on other normative guidance related to assistive technology

| **Title (1.1)** | **Organization (1.2)** | **Year (1.3)** | **Population (2.1)** | **AT functional domain (2.2)** | **Context (2.3)** |
| --- | --- | --- | --- | --- | --- |
| INCOG Recommendations for Management of Cognition Following Traumatic Brain Injury, Part IV: Cognitive Communication | National Institute for Health and Care Excellence (NICE) | 2014 | Individuals with traumatic brain injury (TBI) and cognitive-communication disorders | Communication/speaking | Various healthcare settings, including rehabilitation facilities and community settings |
| Recommendations CODEPEH 2014 for the early detection of delayed hearing loss | Spanish Association of Paediatrics | 2016 | Children, specifically for early detection of hearing loss | Hearing | Primary care settings, early childhood care. |
| IWGDF guidance on the prevention of foot ulcers in at-risk patients with diabetes | International Working Group on the Diabetic Foot (IWGDF) | 2016 | Patients with diabetes at risk for foot ulcers | Mobility, Self-care | Various healthcare settings, including primary care and specialised diabetic foot clinics |
| Vision Screening in Children Aged 6 Months to 5 Years | US Preventive Services Task Force | 2017 | Children aged 6 months to 5 years | Vision | Primary care settings |
| Best Practices for Pressure Ulcer Prevention in the Burn Center | West Penn Hospital, part of Allegheny Health Network, Pittsburgh, Pennsylvania | 2017 | Patients requiring admission to a burn centre | Continence, Mobility | Burn centre settings, including both inpatient burn units and outpatient burn clinics, with a focus on critical care for burn patients. |
| Multidisciplinary recommendations for diagnosis and treatment of foot problems in people with rheumatoid arthritis | Amsterdam Rehabilitation Research Center | 2018 | People with rheumatoid arthritis experiencing foot problems | Mobility, Self-care | Healthcare settings in the Netherlands, involving multidisciplinary teams |
| Integrated care for older people (ICOPE): Guidance for person-centred assessment and pathways in primary care | World Health Organization (WHO) | 2019 | Older adults, specifically those aged 60 years or over, with a focus on those experiencing declines in intrinsic capacity | Hearing, Vision, Continence, Mobility, Cognition, Self-care, Communication/speaking, Environment-modification | Community and primary care settings, applicable in both low- and middle-income countries |
| Rehabilitation of the Laryngectomised Patient. Recommendations of the Spanish Society of Otolaryngology and Head and Neck Surgery | Spanish Society of Otolaryngology and Head and Neck Surgery | 2019 | Patients who have undergone total laryngectomy | Communication/speaking | Various healthcare settings including hospitals and rehabilitation centres |
| Acute care toolkit 3: Acute care for older people living with frailty | Royal College of Physicians | 2020 | Older people (aged 75+) with severe frailty | Hearing, Vision, Continence | Acute Medical Units (AMUs), emergency departments, and community settings |
| Early Diagnosis and Treatment of Unilateral or Asymmetrical Hearing Loss in Children: CODEPEH Recommendations | CODEPEH | 2020 | Children with unilateral or asymmetrical hearing loss | Hearing | Applicable in various healthcare settings, focusing on early diagnosis and treatment |
| Screening for Hearing Loss in Older Adults: US Preventive Services Task Force Recommendation Statement | US Preventive Services Task Force | 2021 | Asymptomatic adults 50 years or older with age-related hearing loss | Hearing | Healthcare settings, including primary care and audiology services |
| Screening for Impaired Visual Acuity in Older Adults | US Preventive Services Task Force | 2022 | Asymptomatic adults 65 years or older who present in primary care without known impaired visual acuity and are not seeking care for vision problems. | Vision | Primary care settings for asymptomatic older adults. |
| The audiologist's guide to hearing aids, personal sound amplification products, hearables, and over-the-counter devices | American academy of audiology | 2022 | All age with or without hearing loss depending on the devices. For PSAP and hearables, only for adults. | Hearing | Audiologist services, and the need for increased awareness of hearing loss and its comorbidities as part of annual primary care examinations |
| WHO-ITU global standard for accessibility of telehealth services | World Health Organization and International Telecommunication Union | 2022 | Persons with disabilities | Vision, Hearing, Communication | Telehealth services in general, particularly for persons with disabilities, in various healthcare settings. |
| WHO Disability-Inclusive Health Services Training Package | World Health Organization (WHO) | 2022 | People with disabilities in the Western Pacific region | Mobility, Communication/speaking, Self-care, Environment-modification | Health facilities, primary health care, local-level clinics, district hospitals |
| Package of Interventions for Rehabilitation, Module 3: Neurological Conditions | World Health Organization (WHO) | 2023 | Adults with neurological conditions such as stroke, Parkinson disease, traumatic brain injury, spinal cord injury, cerebral palsy, and dementia. | Mobility, Self-care, Environment-modification, Continence, Communication/speaking, Cognition | Applicable in various healthcare settings, including hospitals, rehabilitation centres, and community-based care. |
| Package of interventions for rehabilitation: Module 4 Cardiopulmonary conditions | World Health Organization (WHO) | 2023 | Adults with cardiopulmonary conditions, including ischaemic heart disease and chronic obstructive pulmonary disease (COPD) | Mobility, Self-care | Community and healthcare settings |
| Package of interventions for rehabilitation. Module 5. Neurodevelopmental disorders | World Health Organization (WHO) | 2023 | Children, adolescents, and adults with neurodevelopmental disorders, including autism spectrum disorders and intellectual development disorders | Cognition, Vision, Communication, Self-care, Mobility | Various healthcare settings, including community care, primary care, and specialized services |
| Package of interventions for rehabilitation. Module 6. Sensory conditions | World Health Organization (WHO) | 2023 | Children, adolescents, and adults with impairments in visual functions and hearing loss | Vision, Communication, Mobility | Various healthcare settings including community and home environments |
| Package of interventions for rehabilitation. Module 2. Musculoskeletal conditions | World Health Organization (WHO) | 2023 | Adults with musculoskeletal conditions | Mobility, Self-care, Environment-modification | Community-level, primary care, specialist services, multidisciplinary teams |
| Package of Interventions for Rehabilitation - Module 7: Malignant Neoplasm | World Health Organization (WHO) | 2023 | Children and adults with malignant neoplasms | Mobility, Self-care | Rehabilitation settings for individuals with cancer, including community and clinical environments. |
| Package of interventions for rehabilitation. Module 8. Mental health conditions | World Health Organization (WHO) | 2023 | Children, adolescents, and adults with schizophrenia | Communication/speaking | Rehabilitation for schizophrenia takes into consideration an individual's preferences and usually comprises a multifaceted approach that supports individuals to achieve their goals. Focus on social inclusion, community and social life. |
| Hearing aid service delivery approaches for low- and middle-income settings | World Health Organization (WHO) | 2023 | Adults with moderate to severe hearing loss; Children aged 5‚Äì18 years with mild to moderately severe post-lingual hearing loss | Hearing, Communication/speaking | Low- and middle-income settings, community and primary health care facilities |
| EULAR recommendations for the non-pharmacological core management of hip and knee osteoarthritis: 2023 update | European Alliance of Associations for Rheumatology (EULAR) | 2023 | People with hip or knee osteoarthritis | Mobility, Cognition, Continence, Self-care | Various healthcare settings, focusing on individual, community, and policy levels |
